# Psychometric Evaluation of the Chinese Recovering Quality of Life (ReQoL) Outcome Measure and Assessment of Health-Related Quality of Life During the COVID-19 Pandemic

**DOI:** 10.3389/fpsyg.2021.663035

**Published:** 2021-07-28

**Authors:** Richard Huan Xu, Anju Devianee Keetharuth, Ling-ling Wang, Annie Wai-ling Cheung, Eliza Lai-yi Wong

**Affiliations:** ^1^Department of Rehabilitation Sciences, The Hong Kong Polytechnic University, Hong Kong, Hong Kong; ^2^Centre for Health Systems and Policy Research, Jockey Club School of Public Health and Primary Care, The Chinese University of Hong Kong, Hong Kong, Hong Kong; ^3^School of Health and Related Research, The University of Sheffield, Sheffield, United Kingdom; ^4^Blood Transfusion Department, Jinling Hospital, School of Medicine, Nanjing University, Nanjing, China

**Keywords:** health-related quality of life, mental health, recovery, China, validation, ReQoL, psychometric properties

## Abstract

**Objective:**

The primary objective was to translate the Recovering Quality of Life (ReQoL) measures from English to traditional Chinese and assess their psychometric properties in Hong Kong (HK) Chinese population. The secondary objective was to investigate the mental health-related quality of life (HRQoL) of this sample during the coronavirus disease 2019 (COVID-19) pandemic.

**Method:**

Recovering Quality of Life was translated to Traditional Chinese adhering to standard guideline recommended by the official distributors. Five hundred members of the general population were successfully recruited to participate in a telephone-based survey. The following psychometric properties of the ReQoL were evaluated: construct, convergent, and known-group validity and internal consistency and test–retest reliability. The item measurement invariance was assessed on the basis of differential item functioning (DIF). Multiple regression analysis was used to assess the relationship between respondents’ characteristics and mental HRQoL.

**Results:**

Results of confirmatory factor analysis (CFA) supported a two-factor structure of the ReQoL. The ReQoL showed significant correlations with the other mental health, quality of life, and well-being measures, which indicated a satisfactory convergent validity. Known-group validity confirmed that ReQoL is able to differentiate between people with different mental health status. The (Cronbach’s alpha = 0.91 and 0.76 for positive [PF] and negative [NF] factor), and McDonald’s omega of 0.89 (PF = 0.94, NF = 0.82) indicated the ReQoL has good reliability as well as test–retest reliability with an intraclass correlation coefficient of 0.75. Four items showed negligible DIF with respect to age. Respondents who were highly educated and without psychological problems reported a high ReQoL score.

**Conclusion:**

Traditional Chinese ReQoL was shown to be a valid and reliable instrument to assess the recovery-focused quality of life in HK general population. Future studies are needed to appraise its psychometric properties in local people experiencing mental disorders.

## Introduction

Health-related quality of life (HRQoL) captures an individual’s or population’s perceived physical and mental health status over time (Wong et al., 2020). It can provide comprehensive information on the burden of preventable diseases, injuries, and disabilities from the perspective of person-centered care (Yin et al., 2016). Usually, HRQoL is assessed by using patient reported-outcome measures (PROMs), which include multiple items reflecting people’s self-perceived physical and emotional functioning and health status. HRQoL has more traditionally been an important outcome to assess the effectiveness of interventions on people’ physical health (Goldhagen et al., 2016; Xu et al., 2017; Wong E.L.Y. et al., 2019). Recently, however, in evaluating the outcomes of mental care, promoting “recovery,” which reflects the extent to predict the changes in HRQoL (Garner et al., 2014), has drawn increasing professional attention, and emerged as a new paradigm to assess the full journey of people in overcoming the detrimental effects of mental problems (Ellison et al., 2016). Mental health recovery is a self-directed process of healing and transformation (Deegan, 2002), which is promoted through an interaction between individual experience, community environment and social engagement.

Although several PROMs are available to assess the effectiveness of interventions that reduce the symptoms of mental illness, few of them focus on appraising the improvement of mental HRQoL (Keetharuth et al., 2018a). Currently, the EuroQol-five dimension instrument (EQ-5D), a generic preference-based measure, is the most recommended PROM in assessing people’s HRQoL worldwide (Herdman et al., 2011) and has been increasingly used to measure HRQoL in different populations in HK. However, the EQ-5D focused on physical health with only one of five items capturing mental health. In mental health, validity of the EQ-5D is rarely reported but suggests a potentially mixed picture (Brazier, 2010). Although there is evidence that generic instruments are able to reflect the impact of common conditions such as mild to moderate depression and anxiety (Lamers et al., 2006), an increasing number of studies showed conflicting evidence on its validity for patients with schizophrenia, depression, and bipolar (Barton et al., 2009; Papaioannou et al., 2011; Mulhern et al., 2014). Therefore, the Recovering Quality of Life (ReQoL) outcome measure with an emphasis on mental health was constructed to assess the recovery-focused quality of life (Keetharuth et al., 2018b).

Recovering Quality of Life was developed by a team led from The University of Sheffield, United Kingdom and funded by the Department of Health Policy Research Program (ReQoL, 2021). It is a self-completed questionnaire with two versions, ReQoL-20 and ReQoL-10, which contains 20 and 10 mental health items and one physical health item (not included in the scoring), respectively. The ReQoL was developed with significant inputs from service users not only as participants but also as research partners. The psychometric analyses in the development stage were based on data from over 6,450 participants, which significantly increased the face and content validity of the measure (Keetharuth et al., 2018b). A bifactor model of the ReQoL comprising a global factor and two local factors of negative and positive affects was reported by the developers (Keetharuth et al., 2018a). ReQoL has been translated into different languages and shown good reliability and validity (Keetharuth et al., 2018b; Chua et al., 2020; van Aken et al., 2020).

At present, widespread outbreaks of coronavirus disease 2019 (COVID-19) have drastically changed multiple aspects of the people’s lives, with a growing number struggling with several mental health issues (Khan et al., 2020). Mass lockdown, increased rate of unemployment and the fears and uncertainties of the pandemic have not only exacerbated the psychiatric symptoms for patients with mental illnesses, but also affected the general population who may not have previously experienced psychological distress and symptoms of mental illness (Shigemura et al., 2020; Zhang and Ma, 2020). Chew et al. (2020) indicated that these psychological responses affect the well-being of the individual and community, and the impact could persist well after the outbreak. Traditional mental health measures, e.g., General Anxiety Disorder-7 (GAD-7) and Patient Health Questionnaire (PHQ-9), were developed on the basis of meeting clinical criteria and assess the effectiveness of intervention from the perspective of reducing symptoms (Keetharuth et al., 2018b). They cannot be used to reflect the lived experience of general population and measure their HRQoL outcomes. The lack of a well-defined, multidimensional, and psychometrically valid measure of recovery in Hong Kong (HK) has been cited as a potential barrier to providing suitable healthcare for improving their mental HRQoL (Mak et al., 2016). Although the ReQoL was constructed to assess the HRQoL for patients with a broad spectrum of mental illnesses, it could be a useful instrument to capture general wellbeing in the pandemic as well as establishing whether this questionnaire could be used for public health interventions in the general population. Therefore, the primary objective of this study was to translate and culturally adapt the ReQoL from English to Traditional Chinese (ReQoL-TC) and assess its psychometric properties in HK general population. The secondary aim was to investigate the recovery-focused HRQOL of this sample using the ReQoL-TC during the COVID-19 pandemic.

## Materials and Methods

### Translation and Cultural Adaptation

We adhered to standard guidelines “Translation and Linguistic Validation Process for the ReQoL” provided by the official distributors of the ReQoL in translating and culturally adapting the ReQoL in Traditional Chinese (Wild et al., 2005). Dual forward translation was undertaken independently by two professional translators, who were native Chinese speakers but proficient in English. The local research team used both translations to perform the forward translation reconciliation. A revised version was then produced and sent to another two professional translators, who were native English speakers but proficient in Chinese for backward translation independently. The local and ReQoL research team jointly examined the back translation against the original English version to identify any discrepancies, addressed the disputed items, and refined the translation focused on cultural adaption until consensus was achieved by all the research team members.

Cognitive debriefing was conducted with ten members of the general population who were invited to comment on the response options and any wording they found difficult in understanding the ReQoL-TC. Respondents were asked to describe in their own language what the wording meant to them. We paid special attention to the items that we had to adapt and were confident they were understood in the way intended as per the developer’s concept elaboration. After proofreading, the final version of the ReQoL-TC was confirmed.

The local research team has rich experience in the translation and cultural adaption of PROMs and other health outcome-related questionnaires. The eligibility of the translators has been approved by the ReQoL distributor. The results were discussed with the ReQoL developers and one of whom was invited to join local research team to monitor the project and ensure the quality of the development.

### Sample and Data Collection

A random telephone survey of the general population in HK, was carried out by a team of telephone survey professionals in July 2020, following similar recruitment methods by a previous study (Chan et al., 2019). Before the formal survey, a pilot study with ten randomly selected persons was conducted to test the logic of the telephone survey. In order to minimize the sampling error, telephone numbers were first selected randomly from an updated telephone directory as seed numbers. Another three set of numbers were then generated using the randomization of last two digits in order to recruit the unlisted numbers. Duplicate numbers were screened out, and the remaining numbers mixed in a random order to form the final sample. Interviews were carried out by experienced interviewers, between 10:00 and 22:00 on weekdays and other periods including weekends and public holidays should appointments with suitable subjects were arranged. The inclusion criteria for the study were: (1) HK permanent residents; (2) ≥18 years; (3) no cognitive problems; and (4) able to provide informed consent. Upon successful contact with a target household, one qualified member of the household was selected among family members using the last-birthday random selection method (i.e., a respondent in the household who just had their birthday would be selected to participate in the telephone interview). Given a sample size of around 300–500 is believed to have sufficient power to estimate parameters in confirmatory factor analysis (CFA; DeVellis, 2017), in this study, a minimum sample size of 500 was determined. Finally, data from 500 participants were successfully collected. Approximately 72.2% were female, around 60.6% ≥60 years, nearly 64.4% completed the secondary or above education, 62.2% of participants reported no chronic conditions and only 4.4% (*n* = 22) indicated they had visited a psychiatrist within the last 12 months (Table 1). The flowchart of the participant recruitment and selection process is presented in Supplementary Figure 1. Study protocol and informed consent was approved by the institutional review board of The Chinese University of Hong Kong (Ref. ID: SBRE-18-671).

**TABLE 1:** Participants’ characteristics.

	*n*	%
Overall	500	100
**Sex**		
Male	139	27.8
Female	361	72.2
**Age**		
18–49	107	21.4
50–59	90	18.0
60–69	138	27.6
≥70	165	33.0
**Educational level**		
Primary or below	174	34.8
Secondary/post-secondary	216	43.2
Tertiary or above	106	21.2
**Marital status**		
Single	67	13.4
Married	381	76.2
Divorced/widow(er)	50	10.0
**Living status**		
Living alone	52	10.4
Living with families	448	89.6
**Working status**		
Fully employed	139	27.8
Non-employed	179	35.8
Retired	182	36.4
**Government allowance**		
Receiver	177	35.4
Non-receiver	323	64.6
**Personal income per month (HK dollar) [1HKD = 0.13 US dollar]**		
≤5,000	313	62.6
5,001–20,000	93	18.6
≥20,001	45	9.0
Refused	49	9.8
**Chronic conditions**		
Yes	189	37.8
No	311	62.2
**Mental health problem**		
Yes	22	4.4
No	478	95.6

*Non-employed includes respondents who reported they are housewife, students, and unemployed.*

### Measurement

#### Recovering Quality of Life

The ReQoL-TC was used in this study. It comprises 20 mental health items and one physical health item. ReQoL-TC-10 (11 items) comprises the first 10 item of the ReQoL-TC and the physical health item. Of the 20-item ReQoL-TC, 11 are positively worded and nine are negatively worded. All the items are scored on a five-point Likert scale ranging from “None of the time” to “Most or all of the time.” A sum score is calculated by summing the scores of all the items (except for the physical health item), where a higher score indicates a better quality of life.

**TABLE 2:** Fit statistics from confirmatory factor analytic models.

	Bi-factor model: global, negative, positive	Two-factor model: negative and positive
χ^2^	214.7	540.3
Degree of freedom	150	169
*p*-value	<0.001	<0.001
RMSEA (<0.08)	0.029	0.066
SRMR (<0.08)	0.058	0.076
TLI (>0.9)	0.989	0.942
CFI (>0.9)	0.991	0.948

#### General Anxiety Disorder-7

The GAD-7 is a self-rated scale to measure the severity of generalized anxiety disorder. It has seven items, e.g., Feeling nervous, anxious, or on edge, scored from zero (not at all) to three (nearly every day) (Kroenke et al., 2007). The sum score ranges from zero to 21 and the cut-off point for mild, moderate and severe anxiety symptoms are 5, 10, and 15, respectively (Spitzer et al., 2006). The Chinese GAD-7 has been validated (Tong et al., 2015). The Cronbach’s alpha (internal consistency reliability) of the GAD-7 in our sample was 0.93.

#### Depression Anxiety Stress Scales-21 Items

The Depression Anxiety Stress Scales-21 items (DASS-21) measures higher-order mental factor of psychological distress (Lovibond and Lovibond, 1995) over the past week using seven items in each of the domains of depression, anxiety, and stress. Each item, e.g., I found it hard to wind down, has a four-point Likert scale with rating choices ranging between “never applied to oneself” (0) and “very much/most of the time” (3). Final scores for three subscales are calculated by summing the scores for the relevant items (DASS, 2021). The Chinese DASS-21 has been validated (Wang et al., 2016). The Cronbach’s alpha (internal consistency reliability) of subscale depression, anxiety and stress in our sample was 0.75, 0.7, and 0.72, respectively.

#### EQ-5D-5L

The EQ-5D-5L is a generic preference-based measure to estimate people’s HRQoL (Herdman et al., 2011). It has two sections: the descriptive system and the visual analog scale (EQ-VAS). The descriptive system comprises five items (mobility, self-care, usual activities, pain/discomfort, and anxiety/depression) with five levels (from “no problem” to “extreme problem”). Utility scores were calculated using the EQ-5D-5L HK value set (Wong et al., 2018; Wong E.L. et al., 2019). EQ-VAS is a vertical scale used to measure people’s overall health with values between 0 (worst imaginable health) and 100 (best imaginable health) The Cronbach’s alpha (internal consistency reliability) of the EQ-5D-5L (descriptive system) in our sample was 0.82.

#### ICEpop CAPability Measure for Adults

ICEpop CAPability measure for adults (ICECAP-A) is a generic preference-based measure that evaluates an individual’s capability well-being (Al-Janabi et al., 2012). The descriptive system of the ICECAP-A has five items (stability, attachment, autonomy, achievement, and enjoyment) with four response options ranging from “fully capable” to “not capable.” The Chinese ICECAP-A has been validated (Tang et al., 2018). In the absence of value set for the Chinese population, we calculated the sum score of the ICECAP-A by summing the scores of five items, where a higher score represents a poorer capability well-being. The Cronbach’s alpha (internal consistency reliability) of the ICECAP-A in our sample was 0.77.

### Statistical Analysis

#### Construct, Convergent, and Known-Group Validity

Confirmatory factor analysis was used to assess the construct validity. In assessing the dimensionality of the ReQoL-TC, two models were developed in line with the original study (Keetharuth et al., 2018a). The first was a bi-factor model, with one global factor and the two factors contained positively worded and negatively worded items respectively. Second, the two-factor model consisted of the positively worded items and the negatively worded items as two separate factors. The model fit was assessed by the root mean square error of approximation (RMSEA ≤ 0.08), standardized root mean squared residual (SRMR ≤ 0.08), Tucker-Lewis index (TLI ≥ 0.9), and comparative fit index (CFI ≥ 0.9) (DeVellis, 2017). The factor loadings of each item were also checked. For the bi-factor model, we calculated the explained common variance for the global factor to assess its importance relative to the two other factors (Reise et al., 2010). To address the issue of non-normal data, the robust distribution free weighted least squares (WLSMV) estimator was used (Ferrando and Lorenzo-Seva, 2000; Markon, 2019).

Several *a priori* hypotheses about the relationship between the ReQoL-TC and the other HRQoL and mental health instruments were formulated to test the convergent validity of the ReQoL-TC (Keetharuth et al., 2018b; Chua et al., 2020; van Aken et al., 2020) (specific hypotheses are presented in Supplementary Table 1). The strength of the correlation was estimated using Pearson’s correlation coefficient (*r*), where *r* ≥ 0.55 were interpreted as adequate.

The known-group validity was examined by (i) comparing people self-reporting specific mental health conditions versus those who did not; and (ii) using GAD-7 and DASS clinical cut-off points (where a score of <5 on GAD-7 (Spitzer et al., 2006) and depression [≤9], anxiety [≤7], and stress [≤14] on DASS indicate no clinical concerns) (Brumby et al., 2011). While GAD-7 and DASS-21 do not measure aspects of quality of life *per se*, it can be assumed that they define broad groups expected to have different quality of life scores.

#### Item Statistics, Internal Consistency, and Test–Retest Reliability

Feasibility and acceptability of the ReQoL-TC were assessed by the time taken to complete the questionnaire and proportions of missing values of items (respondents were allowed to skip questions) (Xu et al., 2018). The mean, standard deviation (SD), median, and range of the ReQoL-TC (both 20- and 10-item versions) scores were reported. We also calculated ceiling and floor effects, skewness, and kurtosis. Internal consistency reliability of the ReQoL-TC was measured by Cronbach’s alpha (α > 0.7, acceptable), McDonald’s omega (ω > 0.7, acceptable), Guttman’s lambda 4 (λ > 0.8, suitable) (McDonald, 1999), the item-total correlation (>0.5, acceptable) and alpha if an item is dropped (DeVellis, 2017). Selected participants (10%) was invited to take part in another telephone survey two weeks later (only respondents who did not report experiencing any significant life event were invited) to assess the test–retest reliability using intraclass correlation coefficient (ICC > 0.7, acceptable, two-way mixed effects model) (Fleiss, 1999).

#### Differential Item Functioning

Differential item functioning (DIF) with regard to patients’ natural attributes, i.e., gender (male vs. female) and age (regrouped to two groups; G1: < 50 years vs. G2: ≥ 50 years), which were unchanged characteristics, was evaluated (Cherepanov et al., 2010; Roberts et al., 2014). Three ordinal logistic regression models (Model 1: explanatory variable; Model 2: explanatory variable plus vector of group identifiers; and Model 3: explanatory variable multiplied by vector of group identifiers) were developed. The likelihood ratio [χ^2^] was used test to compare the nested models (Robinson et al., 2019). A significant difference (Bonferroni correction was used) between Model 1 and Model 2, and Model 2 and Model 3 indicates the presence of uniform and non-uniform DIF, respectively (Choi et al., 2011). The degree of DIF was assessed on the differences in McFadden’s pseudo-*R*^2^ values (with corresponding effect sizes < 0.13, negligible; 0.13–0.26, moderate; and >0.26, large) (Zumbo, 1999). DIF analyses were conducted for the two dimensions differentiated in the ReQoL-TC.

The univariate (one-way analysis of variance) and multiple analysis (ordinary least squares regression model) was used to show the ReQoL-TC sum scores reported by respondents with different background characteristics, i.e., sex, age, education, marital status, living status, working status, chronic disease status, and mental health condition, respectively. R software was used for all the statistical analyses (R Core Team, 2013). CFA, reliability, ICC and DIF was analyzed using “lavaan,” “psych,” “ICC,” and “lordif” package, respectively. The level of significance was set at *p*-value ≤ 0.05.

## Results

The model fit statistics from the CFA are presented in Table 2. The goodness-of-fit indices indicated an acceptable fit for both the bifactor (χ^2^ = 214.7, degree of freedom [df] = 150, *p* < 0.001, RMSEA = 0.029, SRMR = 0.058, TLI = 0.989, and CFI = 0.991) and the two-factor model (χ^2^ = 540.3, df = 169, *p* < 0.001, RMSEA = 0.066, SRMR = 0.076, TLI = 0.942, and CFI = 0.948) of the ReQoL-TC. The factor loadings for the two-factor model ranged between 0.29 and 0.86. For the bi-factor model, the loadings for the global factor were smaller than 0.3 for 11 out of 20 items (40 factor loadings: 10 for positive factor, 10 for negative factor, and 20 for global factor) and the explained common variance was 51%. Considering the results of CFA and the bi-factor structure reported by the original study, we concluded that the bi-factor model outperformed the two-factor model in this sample of HK general population. The standardized factor loadings for the observed variables of both models are presented in Table 3.

**TABLE 3:** Results of CFA for the ReQoL-TC.

	Item no	Bifactor model standardized loadings			Two-factor model standardized loadings	
Item description		Global	NF	PF	NF	PF
I found it difficult to get started with everyday tasks	r1	0.19	0.39		0.49	
I felt able to trust others	r2	0.34		0.50		0.53
I felt unable to cope	r3	0.26	0.34		0.51	
I could do the things I wanted to do	r4	0.73		0.37		0.82
I felt happy	r5	0.90		0.04		0.74
I thought my life was not worth living	r6	0.09	0.30		0.34	
I enjoyed what I did	r7	0.84		0.25		0.85
I felt hopeful about my future	r8	0.72		0.33		0.80
I felt lonely	r9	0.33		0.32	0.38	
I felt confident in myself	r10	0.66		0.55		0.84
I did things I found rewarding	r11	0.72		0.47		0.86
I avoided things I needed to do	r12	0.01	0.37		0.36	
I felt irritated	r13	0.13	0.61		0.60	
I felt like a failure	r14	0.11	0.30		0.29	
I felt in control of my life	r15	0.64		0.55		0.82
I felt terrified	r16	0.23	0.64		0.58	
I felt anxious	r17	0.01	0.63		0.65	
I had problems with my sleep	r18	0.03	0.47		0.43	
I felt calm	r19	0.20	0.00	0.58		0.42
I found it hard to concentrate	r20	0.02	0.59		0.37	

*NF – factor made up of negatively worded items; PF – factor made up of positively worded items.*

The distribution of the ReQoL-TC sum and factor scores are presented in Table 4. The mean scores (SD) for the ReQoL-TC-20 and the ReQoL-TC-10 were 60.33 (10.52) and 28.54 (6.63), respectively, and no sum score of three scales showed ceiling or floor effect. The internal consistency reliability of ReQoL-TC-20 (α = 0.86 [0.91 and 0.76 for two factors, respectively]) and ReQoL-TC-10 (α = 0.84) was acceptable. The value of ICC confirmed the test–retest reliability of ReQoL-TC-20 (ICC_*overall*_ = 0.75, ICC_*positive*_ = 0.79, and ICC_*negative*_ = 0.71) and ReQoL-TC-10 (ICC = 0.71) was satisfactory. No missing data was identified of ReQoL-TC and the average time to complete the measure was around 5 min indicating a good feasibility and acceptability. The response distribution, and factor-level item-total correlation and alpha if item dropped of the ReQoL-TC are presented in Supplementary Table 3. The sum and factor score distributions of the ReQoL-TC are presented in Supplementary Figure 2.

**TABLE 4:** Score distribution and internal consistency and test–retest reliability of ReQoL-TC.

	ReQoL-20 (scale 0–80)	ReQoL-10 (scale 0–40)	ReQoL (physical item)
**Measure statistics**			
Mean	60.33	28.54	3.57
Standard deviation	10.52	6.63	0.71
Median	61	30	4
Range	22–80	5–40	0–4
Ceiling effect %	0.2	0.6	68.0
Floor effect %	0.2	0.2	0.4
Skewness	−0.42	−0.44	−1.78
Kurtosis	−0.33	−0.58	3.3
**Reliability**			
Overall			
Cronbach’s alpha	0.86	0.84	
McDonald’s omega	0.89	0.86	
Guttman’s lambda 4	0.92	0.88	
ICC (95% C.I.)	0.75 (0.6–0.84)	0.71 (0.54–0.82)	
**Positive domain**			
Cronbach’s alpha	0.91		
McDonald’s omega	0.94		
Guttman’s lambda 4	0.94		
ICC (95% C.I.)	0.79 (0.68–0.87)		
**Negative domain**			
Cronbach’s alpha	0.76		
McDonald’s omega	0.82		
Guttman’s lambda 4	0.86		
ICC (95% C.I.)	0.71 (0.62–0.81)		

*ICC, intraclass correlation coefficient.*

Tables 5, 6 show the result of convergent and known-group validity of the ReQoL-TC. All the correlations between measures were significant and the signs were as expected. Both the ReQoL-TC-20 and ReQoL-TC-10 showed a significant correlation with GAD-7, DASS-21, EQ-5D, and ICECAP-A scores. Most correlation coefficients of the ReQoL-TC-20 were larger than those of the ReQoL-TC-10. The ICECAP-A item of enjoyment (*r* = −0.49) showed the strongest correlation with the sum score of ReQoL-TC-20, followed by the ICECAP-A item of stability (*r* = −0.47) and attachment (*r* = −0.47). The correlation coefficients of the ReQoL-TC-10 with the other measures ranged between 0.23 (GAD-7) and 0.48 (ICECAP-A item of attachment). The results of ANOVA indicated that participants with clinical mental health status showed poorer quality of life than those without, confirming the known-group validity of the ReQoL-TC.

**TABLE 5:** Convergent validity of the ReQoL-TC.

	ReQoL-20			ReQoL-10
	Overall	Positive	Negative	
GAD-7	−0.36***	−0.2***	−0.52***	−0.23***
**DASS-21**				
Depression	−0.44***	−0.21***	−0.62***	−0.33***
Anxiety	−0.38***	−0.16***	−0.56***	−0.26***
Stress	−0.43***	−0.23***	−0.58***	−0.34***
EQ-5D				
Anxiety/Depression item	−0.32***	−0.22***	−0.31***	−0.27***
Utility score	0.3***	0.26***	0.2***	0.3***
EQ-VAS	0.39***	0.3	0.32	0.34***
**ICECAP-A**				
Stability	−0.47***	−0.46***	−0.2***	−0.45***
Attachment	−0.47***	−0.46***	−0.19***	−0.48***
Enjoyment	−0.49***	−0.44***	−0.29***	−0.47***
Achievement	−0.35***	−0.3***	−0.24***	−0.35***
Autonomy	−0.26***	−0.29***	0.06	−0.26***
**ReQoL-20**				
Overall	-	-	-	0.95***
Positive	-	-	0.2***	−

***p < 0.001.

**TABLE 6:** Known-group validity of the ReQoL-TC.

	*N* = 500	Mean (standard deviation)			
		ReQoL-20 (scale 0–80)	ReQoL-20 negative	ReQoL-20 positive	ReQoL-10 (scale 0–40)
**Self-reported mental illness**					
Yes	22	53.09	13.52(3.75)	25.32(8.44)	24.95
No	478	60.66	16.32(5.87)	29.66(8.79)	28.71
*p*-value		<0.001	<0.001	0.02	0.005
**GAD-7**					
No (<5)	437	61.69	20.22(5.2)	30(8.57)	29.13
Mild (5–10)	45	51.49	17.91(4.92)	26.22(8.7)	24.42
Moderate or above (≥10)	18	49.29	12.93(3.13)	25.64(10.3)	24.5
*p*-value		<0.001	<0.001	0.002	<0.001
**DASS-depression**					
Non-clinical (≤9)	472	61.16	21.79(4.88)	29.76(8.8)	28.92
Clinical (> 9)	28	46.36	13.16(3.26)	24.64(7.59)	22.18
*p*-value		< 0.001	<0.001	0.003	< 0.001
**DASS-anxiety**					
Non-clinical (≤7)	470	61.0	20.4(5.35)	29.64(8.87)	28.81
Clinical (>7)	30	49.77	13.21(3.37)	26.8(7.4)	24.4
*p*-value		<0.001	<0.001	0.08	<0.001
**DASS-stress**					
Non-clinical (≤14)	494	60.59	23.83(3.66)	25.59(5.39)	28.66
Clinical (>14)	6	39.17	13.52(3.74)	19.67(8.78)	18.5
*p*-value		<0.001	<0.001	<0.001	<0.001

Factor-level DIF analysis found that items 14 (negative factor) showed both uniform and non-uniform DIF on sex. Another three negatively worded items 6, 10 (uniform), and 9 (non-uniform) showed DIF on age. Two positively worded items 3 (uniform) and 8 (non-uniform) showed DIF on age. Checking the McFadden *R*^2^, the effect size of DIF was negligible for all six items (<0.001–0.06) (Supplementary Table 4).

Results of the univariate and multiple analysis are presented in Table 7. Respondents who were highly educated and living with no psychological problems tended to report a high ReQoL-TC sum score.

**TABLE 7:** Univariable and multiple analysis of the ReQoL-TC.

	Mean (SD)	*p*-value	Coefficient (95% C.I.)
Overall	60.33 (10.52)		
**Sex**			
Male	61.12 (10.88)	0.3	Ref
Female	60.02 (10.38)		−2.04 (−4.29, 0.22)
**Age**			
18–49	60.9 (10.44)	0.6	Ref
50–59	59.92 (10.87)		0.06 (−3.46, 3.58)
60–69	59.48 (9.87)		2.62 (−1.07, 6.32)
≥70	60.89 (10.94)		7.22 (2.53, 11.91)**
**Educational level**			
Primary or below	59.07 (10.64)	<0.001	Ref
Secondary/post-secondary	60.27 (10.52)		2.55 (0.03, 5.08)*
Tertiary or above	63.04 (9.73)		4.09 (0.63, 7.55)*
**Marital status**			
Single	60.85 (8.64)	0.07	Ref
Married	60.66 (10.66)		−0.18 (−3.99, 3.62)
Divorced/widow (er)	57.08 (11.41)		−3.06 (−8.09, 1.97)
**Living status**			
Living alone	56.9 (10.48)	0.01	Ref
Living with families	60.73 (10.47)		1.59 (−2.11, 5.29)
**Working status**			
Fully-employed	60.57 (10.38)	<0.001	Ref
Non-employed	62.09 (9.68)		3.16 (−0.78, 7.1)
Retired	58.41 (11.15)		−3.07 (−7.48, 1.35)
**Government allowance**			
Receiver	59.74 (10.74)	0.4	Ref
Non-receiver	60.65 (10.4)		0.14 (−3.15, 3.43)
**Personal income per month**			
≤5,000	60.48 (10.52)	0.01	Ref
5,001–20,000	58.56 (10.41)		−1.01 (−4.84, 2.81)
≥20,001	63.93 (10.15)		3.43 (−1.34, 8.2)
**Chronic conditions**			
Yes	60.01 (10.58)	0.6	Ref
No	60.52 (10.5)		−0.05 (−2.46, 2.36)
**Mental health problem**			
Yes	53.09 (9.34)	<0.001	Ref
No	60.66 (10.46)		6.96 (2.23, 11.69)*

Non-employed includes respondents who reported they are housewife, students, and unemployed. *p < 0.05; **p < 0.01.

## Discussion

This study presented the development of the Traditional Chinese version of the ReQoL, which exhibited acceptable psychometric properties, in HK general population. The translation process adhered to acceptable international translation standard. A two-factor structure, which separately comprised positively and negatively worded items, was confirmed with an acceptable model fit and factor loadings. We have not found commonly agreed threshold for interpreting the explained common variance. However, previous studies have concluded that scales were sufficiently unidimensional if they obtained ECV values of around 70–80% which is much higher than 51% found in this study (Reise et al., 2013). The ReQoL-TC showed good convergent validity in correlated with other instruments measuring HRQoL, mental health and well-being, and sufficient discriminative power to differentiate people with and without clinical mental health status as defined by GAD-7 and DASS-21. Additionally, the internal consistency and test–retest reliability of the ReQoL-TC were satisfactory for both positive wording and negative wording factors, and no ceiling or floor effect was detected. Further, a significant and strong correlation between ReQoL-TC-20 and ReQoL-TC-10 was identified. In general, the results of this study confirmed that two-factor ReQoL-TC is a valid and reliable instrument with good acceptability and feasibility to assess the recovery-focused quality of life for HK general population and can be used in research settings.

Although no ceiling or floor effects were detected on the sum score of ReQoL-TC, score distribution of some negatively worded items were severely skewed. Approximately 92 and 89% of participants chose the option “Never” for item “*I felt like a failure*” and “*I thought my life was not* worth living.” This is in line with the findings from the original United Kingdom study where 33 and 51% of patients indicated never having any concerns on those two aspects. Additionally, item “*I felt in control of my life*” and item “*I felt calm*” showed a measure of floor effect with 31 and 25% of participants indicating they could control their life or feel calm, respectively, which was not reported in the original study. These findings should be interpreted with caution as our survey was completed during the COVID-19 pandemic. Given several studies have confirmed that pandemic undoubtedly negatively impact on people’s mental health and well-being (Burgess, 2020; Galea et al., 2020), the “COVID bias” might have affected the people’s response in our study. Post-pandemic assessments are needed in the future.

The ReQoL-TC has a short version and a longer version to serve the different settings where recovery-focused quality of life is measured. Despite the internal consistency and test–retest reliability for both versions being satisfactory in this study, the Cronbach’s alpha was lower than that reported by the studies in United Kingdom (ReQoL-20:0.93 and ReQoL-10:0.87) and Netherlands (ReQoL-20:0.94 and ReQoL-10:0.9) (Keetharuth et al., 2018b; van Aken et al., 2020). We found the mean sum score of the ReQoL-TC-10 was lower than that of the half of ReQoL-TC-20 sum score (scale 0–40). This difference might be because participants tend to score higher on those items with negative wording. ReQoL-TC-10 only contains four out of 11 items with negative wordings. However, previous studies indicated that a measure contains a mixture of positive and negative items is a crucial element as people with mental health difficulties identified issues that both enhanced or depleted their quality of life (Crawford et al., 2011; Keetharuth et al., 2018b). The psychometric performance of two versions of ReQoL-TC should be separately assessed in the future.

In our sample, compared with the ReQoL-TC-10, the ReQoL-TC-20 showed a closer relationship with the mental health-related measures, i.e., the GAD-7 and DASS-21. This is possibly the case because of the pandemic with more people experiencing mental health difficulties. This finding should be interpreted with caution because only 500 members of the general population were included in this study. However, overall, we could expect ReQoL-TC-10, by virtue of its brevity, to be more practical measuring the recovery-focused quality of life for general population. For individuals with mental illness, the ReQoL-TC-20 could be more appropriate due to its comprehensiveness. Future studies with both general population and individuals with mental problems are needed to investigate the generatability of findings in this study. Further, in line with the findings of van Aken et al.’s (2020) study, both ReQoL-20 and ReQoL-10 showed a significant but not strong correlation with the EQ-5D utility score. It is not surprising that, as Papaioannou et al. (2013) also indicated in their study, for patients with personality disorders, the EQ-5D is suitable to assess patients’ HRQoL, but lacks the content validity to fully reflect the impact of the condition. Brazier also indicated that the EQ-5D appears to perform acceptably well in depression and personality disorder, but less well in anxiety, schizophrenia and bipolar disorder (Brazier et al., 2014). Furthermore, the ReQoL-TC sum score strongly correlated with ICECAP-A item of stability, attachment and enjoyment supported that ReQoL-TC can capture people’ recovery-focused quality of life as it is intended to measure (Leamy et al., 2011), instead of assessing the effect of the reduction in symptoms alone.

The associations of positively and negatively worded factors of the ReQoL-TC with the other HRQoL and mental health measures showed a mixed picture. The items of negative wordings presented a strong correlation with the measures investigating individual’s mental health status. However, the items of positive wordings factor showed a strong correlation with quality of life and well-being measures. Previous studies have indicated the potential impact of using negative or positive wordings in assessing individual’s psychological attributes. For example, a study examined the Rosenberg Self-Esteem scale, has demonstrated the existence of method effects associated with negatively and/or positively worded items (Schönberger and Ponsford, 2009). Wouters et al. (2012) also indicated the importance of evaluating wording effects when examining the factor structure of the HADS in vulnerable patient groups. However, few of them directly exhibited a significant association between items with positive wordings and respondents’ well-being. Further research is needed to investigate the effect of negatively and positively worded items of the ReQoL-TC on the other Chinese population’s health, and which socio-demographic and personality characteristics are associated with such response style.

Regarding the structure of the ReQoL-TC, Keetharuth et al.’s (2018b) original study reported a bi-factor model – a global factor and separate factors for the positively and negatively worded items. This finding was not fully supported in our study (low factor loadings for the global factor and a low explained common variance), despite the satisfactory goodness-of-fit indices. However, both two studies confirmed the presence of two factors – positively worded and negatively worded items – of the ReQoL. Moreover, our model fit was average as this might be as a result of the survey bias in terms of the interviewer administered questionnaire, the survey population and the influence of the COVID-19 pandemic. Considering the difference in the populations of the two studies (patient in the United Kingdom vs. general population in HK) and the goodness-of-fit of two models were satisfactory, we do not suggest rejecting the two-factor model. However, further assessments are needed to explore the structure of the ReQoL-TC in both HK general population or individuals experiencing mental illness. The implication of the two factors is that a sum score may not be generated for the ReQoL-TC for this population and the scores of positive and negative affect will have to be kept separate. Moreover, several items (four negative and one positive wording items) showed significant DIF on age and sex, respectively, despite the effect size of DIF was negligible.

Attention should be paid to the item “*I felt like a failure (item 14)*,” which showed several psychometric problems, such as both uniform and non-uniform DIF, a low factor loading (0.29) and strong skewness (−5.32). This might be because of two possible reasons. First, “failure” is a very negative word in the Chinese culture and people usually show less willingness to use that word to describe themselves or the others (Bedford and Hwang, 2003), thus, in this study, more than 90% of respondents selected “none of the time”. Second, the ReQoL-TC was developed based on inputs from individuals with mental health problems, however, in this study, all respondents were members of the general population, thus, negative wording items, such as “failure” item were problematic to some extent. However, considering this was the first study to assess the validity and reliability of the ReQoL-TC and only 500 members of the general population was surveyed, we retained all the items and did not recommend for any to be dropped at this stage.

Several limitations should be addressed. First, all participants in this study were recruited through a telephone-based survey. This might lead to several bias pertaining to data collection and quality, e.g., participants may not have understood the questions as they could not read them (interview bias). Hence, other forms, such as face-to-face or online survey, should be used in future studies. Second, our sample is not representative of the HK general population as few of them were young or with high income, which might affect the validity and reliability of the ReQoL-TC. Third, while we used the guidelines provided by the developers to adapt the ReQoL for the HK population, we are aware that there are newer guidelines on cultural adaptation (Hernandez et al., 2020). Moreover, despite the original ReQoL has been translated and adapted to HK Chinese population, the adaptation may not be directly used to compare between two cultures because no measurement equivalence between the original and the adapted forms was carried out. Further investigations are needed to independently assess the psychometric properties of the ReQoL-TC-10 in another sample of local population.

## Conclusion

This study confirmed that the ReQoL-TC has sound psychometric properties in a sample of HK general population. It demonstrates good face and content validity, satisfactory convergent and discriminatory validity as well as adequate internal consistency and test–retest reliability. A bi-factor structure of the ReQoL-TC with one positive wording, one negative wording and a global factor was confirmed. This study also investigated the recovery-focused HRQoL using the newly developed ReQoL-TC in HK general population during the COVID-19 pandemic. Although we showed that ReQoL-TC is suitable for use in HK general population, future validation work should be carried out to investigate the performance of the ReQoL-TC in individuals with mental health problems. We also intend to develop a set of preference weights preference-based ReQoL-TC to calculate quality adjusted life years to support the economic evaluation in improving people’s mental HRQoL.

## Data Availability Statement

The raw data supporting the conclusions of this article will be made available by contacting correspondence author, without undue reservation.

## Ethics Statement

The studies involving human participants were reviewed and approved by the institutional review board of The Chinese University of Hong Kong (Ref. ID: SBRE-18-671). The patients/participants provided their written informed consent to participate in this study.

## Author Contributions

RX: study concept and design, data analysis and interpretation, software, writing-original draft, and writing–review and editing. AK: study concept and design, data analysis and interpretation, and writing–review and editing. L-LW: software, visualization, and writing–review and editing. AW-LC: provision of study materials and patients, collection and assembly of data, and writing–review and editing. EL-YW: study concept and design, provision of study materials and patients, collection and assembly of data, supervision, and writing–review and editing. All authors contributed to the article and approved the submitted version.

## Conflict of Interest

The authors declare that the research was conducted in the absence of any commercial or financial relationships that could be construed as a potential conflict of interest.

## Publisher’s Note

All claims expressed in this article are solely those of the authors and do not necessarily represent those of their affiliated organizations, or those of the publisher, the editors and the reviewers. Any product that may be evaluated in this article, or claim that may be made by its manufacturer, is not guaranteed or endorsed by the publisher.

## References

[B1] Al-Janabi H. Flynn T. N. Coast J. (2012). Development of a self-report measure of capability wellbeing for adults: the ICECAP-A. *Qual. Life Res.* 21 167–176. 10.1007/s11136-011-9927-2 21598064 PMC3254872

[B2] Barton G. R. Hodgekins J. Mugford M. Jones P. B. Croudace T. Fowler D. (2009). Measuring the benefits of treatment for psychosis: validity and responsiveness of the EQ–5D. *Br. J. Psychiatry* 195 170–177. 19648552 10.1192/bjp.bp.108.057380

[B3] Bedford O. Hwang K.-K. (2003). Guilt and shame in chinese culture: a cross-cultural framework from the perspective of morality and identity. *J. Theory Soc. Behav.* 33 127–144. 10.1111/1468-5914.00210

[B4] Brazier J. (2010). Is the EQ-5D fit for purpose in mental health? *Br. J. Psychiatry* 197:348. 10.1192/bjp.bp.110.082453 21037210

[B5] Brazier J. Connell J. Papaioannou D. Mukuria C. Mulhern B. Peasgood T. (2014). A systematic review, psychometric analysis and qualitative assessment of generic preference-based measures of health in mental health populations and the estimation of mapping functions from widely used specific measures. *Health Technol. Assess.* 18 7–8. 10.3310/hta18340 24857402 PMC4781324

[B6] Brumby S. Chandrasekara A. McCoombe S. Torres S. Kremer P. Lewandowski P. (2011). Reducing psychological distress and obesity in Australian farmers by promoting physical activity. *BMC Public Health* 11:362. 10.1186/1471-2458-11-362 21600058 PMC3118243

[B7] Burgess R. (2020). COVID-19 mental-health responses neglect social realities. *Nature* 10.1038/d41586-020-01313-9 32366975

[B8] Chan C. W. H. Wong M. H. H. Chan K. W. Chow A. Y. M. Lo R. S. Y. (2019). Prevalence, perception, and predictors of advance directives among Hong Kong Chinese: a population-based survey. *Int. J. Environ. Res. Public Health* 16:365. 10.3390/ijerph16030365 30696082 PMC6388376

[B9] Cherepanov D. Palta M. Fryback D. G. Robert S. A. (2010). Gender differences in health-related quality-of-life are partly explained by Sociodemographic and socioeconomic variation between adult men and women in the US: evidence from four US nationally representative data sets. *Qual. Life Res.* 19 1115–1125. 10.1007/s11136-010-9673-x 20496168 PMC2940034

[B10] Chew Q. H. Wei K. C. Vasoo S. Chua H. C. Sim K. (2020). Narrative synthesis of psychological and coping responses towards emerging infectious disease outbreaks in the general population: practical considerations for the COVID-19 pandemic. *Singapore Med. J.* 61 350–356. 10.11622/SMEDJ.2020046 32241071 PMC7926608

[B11] Choi S. W. Gibbons L. E. Crane P. K. (2011). Lordif: an R package for detecting differential item functioning using iterative hybrid ordinal logistic regression/ itm response theory and monte carlo simulations. *J. Stat. Softw.* 39 1–30. 10.1002/jcp.22063.Downregulation PMC3093114 21572908

[B12] Chua Y. C. Wong H. H. Abdin E. Vaingankar J. Shahwan S. Cetty L. (2020). The Recovering Quality of Life 10-item (ReQoL-10) scale in a first-episode psychosis population: validation and implications for patient-reported outcome measures (PROMs). *Early Interv. Psychiatry* 1–9. 10.1111/eip.13050 33058560

[B13] Crawford M. J. Robotham D. Thana L. Patterson S. Weaver T. Barber R. (2011). Selecting outcome measures in mental health: the views of service users. *J. Ment. Health* 20 336–346. 10.3109/09638237.2011.577114 21770782

[B14] DASS (2021). *Depression, Anxiety, Stress Scales (DASS).* Available online at: http://www2.psy.unsw.edu.au/dass/ (accessed April 19, 2021).

[B15] Deegan P. E. (2002). Recovery as a self-directed process of healing and transformation. *Occup. Ther. Ment. Health* 17 5–21. 10.1300/J004v17n03_02

[B16] DeVellis R. F. (2017). *Scale Development: Theory and Applications*, 4the Edn. Los Angeles: SAGE.

[B17] Ellison M. L. Belanger L. K. Niles B. L. Evans L. C. Bauer M. S. (2016). Explication and definition of mental health recovery: a systematic review. *Adm. Policy Ment. Health Ment. Health Serv. Res.* 45 91–102. 10.1007/s10488-016-0767-9 27709376

[B18] Ferrando P. J. Lorenzo-Seva U. (2000). Universitat Rovira i Virgili unrestricted versus restricted factor analysis of multidimensional test items: some aspects of the problem and some suggestions. *Psicológica* 21 301–323.

[B19] Fleiss J. L. (1999). *The Design and Analysis of Clinical Experiments.* New York, NY: Wiley.

[B20] Galea S. Merchant R. M. Lurie N. (2020). The mental health consequences of COVID-19 and physical distancing: the need for prevention and early intervention. *JAMA Intern. Med.* 180:817. 10.1001/jamainternmed.2020.1562 32275292

[B21] Garner B. R. Scott C. K. Dennis M. L. Funk R. R. (2014). The relationship between recovery and health-related quality of life. *J. Subst. Abuse Treat.* 47 293–298. 10.1016/j.jsat.2014.05.006 25012552 PMC4138291

[B22] Goldhagen J. Fafard M. Komatz K. Eason T. Livingood W. C. (2016). Erratum to: community-based pediatric palliative care for health related quality of life, hospital utilization and costs lessons learned from a pilot study. *BMC Palliat. Care* 15:73. 10.1186/s12904-016-0138-z 27487770 PMC4971636

[B23] Herdman M. Gudex C. Lloyd A. Janssen M. F. Kind P. Parkin D. (2011). Development and preliminary testing of the new five-level version of EQ-5D (EQ-5D-5L). *Qual. Life Res.* 20 1727–1736. 10.1007/s11136-011-9903-x 21479777 PMC3220807

[B24] Hernandez A. Hidalgo D. Hambleton R. K. Gomez-Benito J. (2020). International Test Commission guidelines for test adaptation: a criterion checklist/Directrices de la Comisidn International de Test para la adaptation de test: un listado de verification. *Psicothema* 32 390–398. 10.7334/psicothema2019.306 32711675

[B25] Keetharuth A. D. Bjorner J. B. Barkham M. Browne J. Croudace T. Brazier J. (2018a). Exploring the item sets of the Recovering Quality of Life (ReQoL) measures using factor analysis. *Qual. Life Res.* 28 1005–1015. 10.1007/s11136-018-2091-1 30578454 PMC6439178

[B26] Keetharuth A. D. Brazier J. B. Connell J. Bjorner J. B. Carlton T. Buck L. (2018b). Recovering Quality of Life (ReQoL): a new generic self-reported outcome measure for use with people experiencing mental health difficulties. *Br. J. Psychiatry* 212 42–49. 10.1192/bjp.2017.10 29433611 PMC6457165

[B27] Khan K. S. Mamun M. A. Griffiths M. D. Ullah I. (2020). The mental health impact of the COVID-19 pandemic across different cohorts. *Int. J. Ment. Health Addict.* 3 1–7. 10.1007/s11469-020-00270-8 32837440 PMC7347045

[B28] Kroenke K. Spitzer R. L. Williams J. B. Monahan P. O. Löwe B. (2007). Anxiety disorders in primary care: prevalence, impairment, comorbidity, and detection. *Ann. Intern. Med.* 146:317. 10.7326/0003-4819-146-5-200703060-00004 17339617

[B29] Lamers L. M. Bouwmans C. A. van Straten A. Donker M. C. Hakkaart L. (2006). Comparison of EQ-5D and SF-6D utilities in mental health. *Health Econ.* 15 1229–1236. 10.1002/hec.1125 16625671

[B30] Leamy M. Bird V. Le Boutillier C. Williams J. Slade M. (2011). Conceptual framework for personal recovery in mental health: systematic review and narrative synthesis. *Br. J. Psychiatry* 199 445–452. 10.1192/bjp.bp.110.083733 22130746

[B31] Lovibond S. H. Lovibond P. F. (1995). *Manual for the Depression Anxiety Stress Scales*, 2nd Edn, Sydney, NSW: Psychology Foundation of Australia.

[B32] Mak W. W. S. Chan R. C. H. Pang I. H. Y. Chung N. Y. L. Yau S. S. W. Tang J. P. S. (2016). Effectiveness of Wellness Recovery Action Planning (WRAP) for Chinese in Hong Kong. *Am. J. Psychiatr. Rehabil.* 19 235–251. 10.1080/15487768.2016.1197859

[B33] Markon K. E. (2019). Bifactor and hierarchical models: specification, inference, and interpretation. *Annu. Rev. Clin. Psychol.* 15 51–69. 30649927 10.1146/annurev-clinpsy-050718-095522

[B34] McDonald R. P. (1999). *Test Theory?: A Unified Treatment.* Mahwah, NJ: L. Erlbaum Associates.

[B35] Mulhern B. Mukuria C. Barkham M. Knapp M. Byford S. Soeteman D. (2014). Using generic preference-based measures in mental health: psychometric validity of the EQ-5D and SF-6D. *Br. J. Psychiatry* 205 236–243. 24855127 10.1192/bjp.bp.112.122283

[B36] Papaioannou D. Brazier J. Parry G. (2011). How valid and responsive are generic health status measures, such as EQ-5D and SF-36, in Schizophrenia? A systematic review. *Value Health* 14 907–920. 21914513 10.1016/j.jval.2011.04.006 PMC3179985

[B37] Papaioannou D. Brazier J. Parry G. (2013). How to measure quality of life for cost-effectiveness analyses of personality disorders: a systematic review. *J. Pers. Disord.* 27 383–401. 10.1521/pedi_2013_27_075 23445474

[B38] R Core Team (2013). *A Language and Environment for Statistical Computing.* Vienna: R Foundation for Statistical Computing.

[B39] Reise S. P. Bonifay W. E. Haviland M. G. (2013). Scoring and modeling psychological measures in the presence of multidimensionality. *J. Pers. Assess.* 95 129–140. 10.1080/00223891.2012.725437 23030794

[B40] Reise S. P. Moore T. M. Haviland M. G. (2010). Bifactor models and rotations: exploring the extent to which multidimensional data yield univocal scale scores. *J. Pers. Assess.* 92 544–559. 10.1080/00223891.2010.496477 20954056 PMC2981404

[B41] ReQoL (2021). *ReQoL: OVERVIEW.* Available online at: https://www.reqol.org.uk/p/overview.html (accessed April 19, 2021).

[B42] Roberts J. Lenton P. Keetharuth A. D. Brazier J. (2014). Quality of life impact of mental health conditions in England: results from the adult psychiatric morbidity surveys. *Health Qual. Life Outcom.* 12:6. 10.1186/1477-7525-12-6 24422899 PMC3901021

[B43] Robinson M. Johnson A. M. Walton D. M. MacDermid J. C. (2019). A comparison of the polytomous Rasch analysis output of RUMM2030 and R (ltm/eRm/TAM/lordif). *BMC Med. Res. Methodol.* 19:36. 10.1186/s12874-019-0680-5 30786868 PMC6381688

[B44] Schönberger M. Ponsford J. (2009). The factor structure of the hospital anxiety and depression scale in individuals with traumatic brain injury. *Psychiatry Res.* 179 342–349. 10.1016/j.psychres.2009.07.003 20483471

[B45] Shigemura J. Ursano R. J. Morganstein J. C. Kurosawa M. Benedek D. M. (2020). Public responses to the novel 2019 coronavirus (2019-nCoV) in Japan: mental health consequences and target populations. *Psychiatry Clin. Neurosci.* 74 281–282. 10.1111/pcn.12988 32034840 PMC7168047

[B46] Spitzer R. L. Kroenke K. Williams J. B. Löwe B. (2006). A brief measure for assessing generalized anxiety disorder: the GAD-7. *Arch. Intern. Med.* 166 1092–1097. 16717171 10.1001/archinte.166.10.1092

[B47] Tang C. Xiong Y. Wu H. Xu J. (2018). Adaptation and assessments of the Chinese version of the ICECAP-A measurement. *Health Qual. Life Outcom.* 16 11–45. 10.1186/s12955-018-0865-3 29530092 PMC5848585

[B48] Tong X. An D. McGonigal A. Park S. P. Zhou D. (2015). Validation of the Generalized Anxiety Disorder-7 (GAD-7) among Chinese people with epilepsy. *Epilepsy Res.* 120 31–36. 26709880 10.1016/j.eplepsyres.2015.11.019

[B49] van Aken B. C. de Beurs E. Mulder C. L. van der Feltz-Cornelis C. M. (2020). The dutch recovering quality of life questionnaire (ReQoL) and its psychometric qualities. *Eur. J. Psychiatry* 34 99–107.

[B50] Wang K. Shi H. S. Geng F. L. Zou L. Q. Tan S. P. Wang Y. (2016). Cross-cultural validation of the depression anxiety stress scale-21 in China. *Psychol. Assess.* 28 e88–e100. 26619091 10.1037/pas0000207

[B51] Wild D. Grove A. Martin M. Eremenco S. McElroy S. Verjee-Lorenz A. (2005). Principles of good practice for the translation and cultural adaptation process for patient-reported Outcomes (PRO) measures: report of the ISPOR task force for translation and cultural adaptation. *Value Health* 8 94–104. 10.1111/j.1524-4733.2005.04054.x 15804318

[B52] Wong E. L. Y. Ramos-Goñi J. M. Cheung A. W. L. Wong A. Y. K. Rivero-Arias O. (2018). Assessing the use of a feedback module to model EQ-5D-5L health states values in Hong Kong. *Patient* 11 235–247. 10.1007/s40271-017-0278-0 29019161 PMC5845074

[B53] Wong E. L. Y. Xu R. H. Cheung A. W. L. (2019). Health-related quality of life among patients with hypertension: population-based survey using EQ-5D-5L in Hong Kong SAR, China. *BMJ Open* 9:e032544. 10.1136/bmjopen-2019-032544 31562165 PMC6773333

[B54] Wong E. L. Y. Xu R. H. Cheung A. W. L. (2020). Measurement of health-related quality of life in patients with diabetes mellitus using EQ-5D-5L in Hong Kong, China. *Qual. Life Res.* 29 1913–1921. 10.1186/1477-7525-8-18 32140920 PMC7295714

[B55] Wong E. L. Cheung A. W. Wong A. Y. Xu R. H. Ramos-Goñi J. M. Rivero-Arias O. (2019). Normative profile of health-related quality of life for Hong Kong general population using preference-based instrument EQ-5D-5L. *Value Health* 22 916–924. 10.1016/j.jval.2019.02.014 31426933

[B56] Wouters E. Booysen F. R. Ponnet K. Baron van Loon F. (2012). Wording effects and the factor structure of the hospital anxiety & depression scale in HIV/AIDS patients on antiretroviral treatment in south Africa. *PLoS One* 7:e34881. 10.1371/journal.pone.0034881 22536338 PMC3335020

[B57] Xu R. H. Cheung A. W. L. Wong E. L. (2017). Examining the health-related quality of life using EQ-5D-5L in patients with four kinds of chronic diseases from specialist outpatient clinics in Hong Kong SAR, China. *Patient Prefer Adheren.* 11 1565–1572. 10.2147/PPA.S143944 28979104 PMC5602472

[B58] Xu R. H. Cheung A. W. L. Wong E. L. Y. (2018). Development and validation of an instrument to measure patient engagement in Hong Kong Special Administrative Region, China. *Patient Prefer Adheren.* 12 1667–1675. 10.2147/PPA.S171026 30233147 PMC6129019

[B59] Yin S. Njai R. R. Barker L. Siegel R. Z. Liao Y. (2016). Summarizing health-related quality of life (HRQOL): development and testing of a one-factor model. *Popul. Health Metr.* 14:22. 10.1186/s12963-016-0091-3 27408606 PMC4940947

[B60] Zhang Y. Ma Z. F. (2020). Impact of the COVID-19 pandemic on mental health and quality of life among local residents in liaoning province, China: a cross-sectional study. *Int. J. Environ. Res. Public Health* 17:2381. 10.3390/ijerph17072381 32244498 PMC7177660

[B61] Zumbo B. D. (1999). *A Handbook on the Theory and Methods of Differential Item Functioning (DIF): Logistic Regression Modeling as a Unitary Framework for Binaryand Likert-Type (Ordinal) Item Scores.* Ottawa: Directorate of Human Resources Research and Evaluation National Defense.

