# Supplementary material for: Psychometric Evaluation of the Chinese Recovering Quality of Life (ReQoL) Outcome Measure and Assessment of Health-Related Quality of Life During the COVID-19 Pandemic
Source: Front Psychol. 2021 Jul 28;12:663035. doi: 10.3389/fpsyg.2021.663035 (PMC8356898; doi:10.3389/fpsyg.2021.663035)
Supplement: Supplementary file 1 [file Table_1.DOCX]

Table S1 Hypotheses of correlations between ReQoL and other measures

|  | **Relationship** | **Targeted measurements** |
| --- | --- | --- |
| ReQoL sum score | Significant and negative correlation with | DASS-depression |
| ReQoL sum score | Significant and negative correlation with | DASS-anxiety |
| ReQoL sum score | Significant and negative correlation with | DASS-stress |
| ReQoL sum score | Significant and negative correlation with | ICECAP-A: Stability |
| ReQoL sum score | Significant and negative correlation with | ICECAP-A: Attachment |
| ReQoL sum score | Significant and negative correlation with | ICECAP-A: Enjoyment |
| ReQoL sum score | Significant and negative correlation with | ICECAP-A: Achievement |
| ReQoL sum score | Significant and negative correlation with | ICECAP-A: Autonomy |
| ReQoL sum score | Significant and negative correlation with | EQ-5D Anxiety/depression item |
| ReQoL sum score | Significant and negative correlation with | GAD-7 |
| ReQoL sum score | Significant and positive correlation with | EQ-5D utility score |
| ReQoL sum score | Significant and positive correlation with | EQ-5D EQ-VAS score |

Table S2 Polychoric correlations between ReQoL items

|  | r1 | r2 | r3 | r4 | r5 | r6 | r7 | r8 | r9 | r10 | r11 | r12 | r13 | r14 | r15 | r16 | r17 | r18 | r19 |
| --- | --- | --- | --- | --- | --- | --- | --- | --- | --- | --- | --- | --- | --- | --- | --- | --- | --- | --- | --- |
| r1 | 1 |  |  |  |  |  |  |  |  |  |  |  |  |  |  |  |  |  |  |
| r2 | -0.03 | 1 |  |  |  |  |  |  |  |  |  |  |  |  |  |  |  |  |  |
| r3 | 0.34 | 0.05 | 1 |  |  |  |  |  |  |  |  |  |  |  |  |  |  |  |  |
| r4 | 0.15 | 0.54 | 0.24 | 1 |  |  |  |  |  |  |  |  |  |  |  |  |  |  |  |
| r5 | 0.31 | 0.36 | 0.31 | 0.71 | 1 |  |  |  |  |  |  |  |  |  |  |  |  |  |  |
| r6 | 0.4 | 0.02 | 0.22 | 0.08 | 0.23 | 1 |  |  |  |  |  |  |  |  |  |  |  |  |  |
| r7 | 0.19 | 0.42 | 0.31 | 0.8 | 0.77 | 0.08 | 1 |  |  |  |  |  |  |  |  |  |  |  |  |
| r8 | 0.2 | 0.48 | 0.27 | 0.64 | 0.68 | 0.18 | 0.72 | 1 |  |  |  |  |  |  |  |  |  |  |  |
| r9 | 0.34 | 0.02 | 0.3 | 0.32 | 0.32 | 0.42 | 0.36 | 0.28 | 1 |  |  |  |  |  |  |  |  |  |  |
| r10 | 0.18 | 0.5 | 0.19 | 0.74 | 0.61 | 0.2 | 0.74 | 0.7 | 0.42 | 1 |  |  |  |  |  |  |  |  |  |
| r11 | 0.2 | 0.44 | 0.22 | 0.75 | 0.66 | 0.1 | 0.77 | 0.75 | 0.32 | 0.8 | 1 |  |  |  |  |  |  |  |  |
| r12 | 0.52 | -0.14 | 0.23 | -0.03 | 0.16 | 0.5 | -0.02 | 0.03 | 0.34 | 0.08 | 0.02 | 1 |  |  |  |  |  |  |  |
| r13 | 0.44 | -0.02 | 0.32 | 0.08 | 0.21 | 0.38 | 0.14 | 0.13 | 0.41 | 0.13 | 0.08 | 0.4 | 1 |  |  |  |  |  |  |
| r14 | 0.43 | 0.07 | 0.39 | 0.14 | 0.18 | 0.68 | 0.04 | 0.14 | 0.49 | 0.23 | 0.13 | 0.5 | 0.54 | 1 |  |  |  |  |  |
| r15 | 0.12 | 0.46 | 0.22 | 0.7 | 0.62 | 0.12 | 0.74 | 0.72 | 0.36 | 0.77 | 0.77 | -0.02 | 0.08 | 0.14 | 1 |  |  |  |  |
| r16 | 0.38 | 0.05 | 0.37 | 0.15 | 0.38 | 0.41 | 0.21 | 0.2 | 0.32 | 0.2 | 0.12 | 0.39 | 0.61 | 0.46 | 0.15 | 1 |  |  |  |
| r17 | 0.33 | 0 | 0.36 | 0 | 0.13 | 0.4 | -0.01 | 0.07 | 0.26 | 0 | -0.05 | 0.36 | 0.62 | 0.47 | -0.1 | 0.81 | 1 |  |  |
| r18 | 0.25 | -0.05 | 0.29 | 0 | 0.1 | 0.38 | 0.03 | 0.11 | 0.24 | 0.02 | 0.01 | 0.26 | 0.32 | 0.49 | 0.04 | 0.4 | 0.41 | 1 |  |
| r19 | 0.07 | 0.38 | 0.07 | 0.36 | 0.19 | 0.05 | 0.32 | 0.32 | 0.36 | 0.46 | 0.4 | -0.09 | 0.17 | 0.17 | 0.54 | 0.02 | -0.02 | -0.11 | 1 |
| r20 | 0.38 | -0.02 | 0.34 | 0.03 | 0.18 | 0.43 | -0.03 | -0.05 | 0.2 | 0.04 | -0.08 | 0.47 | 0.43 | 0.44 | -0.1 | 0.54 | 0.5 | 0.51 | -0.16 |

Table S3 Item statistics of the ReQoL

| **Item** | **Response level** | | | | | **Item-total correlation** | **alpha if item deleted** | **Skewness** | **kurtosis** |
| --- | --- | --- | --- | --- | --- | --- | --- | --- | --- |
|  | **0** | **1** | **2** | **3** | **4** |  |  |  |  |
| I found it difficult to get started with everyday tasks | 0.6% | 1.8% | 4.4% | 17.0% | 76.2% | 0.51 | 0.86 | -2.49 | 6.78 |
| I felt able to trust others | 12.6% | 7.8% | 20.2% | 44.0% | 15.4% | 0.64 | 0.85 | -0.74 | -0.37 |
| I felt unable to cope | 0.4% | 2.0% | 9.4% | 28.4% | 59.8% | 0.49 | 0.86 | -1.43 | 1.85 |
| I could to the things I wanted to do | 6.2% | 14.0% | 18.8% | 40.0% | 21.0% | 0.83 | 0.84 | -0.61 | -0.47 |
| I felt happy | 13.4% | 10.4% | 23.2% | 34.8% | 18.2% | 0.74 | 0.84 | -0.51 | -0.74 |
| I thought my life was not worth living | 1.0% | 1.2% | 2.4% | 6.8% | 88.6% | 0.46 | 0.86 | -3.97 | 17 |
| I enjoyed what I did | 8.0% | 15.8% | 17.2% | 42.4% | 16.6% | 0.84 | 0.84 | -0.58 | -0.61 |
| I felt hopeful about my future | 21.8% | 20.8% | 20.6% | 26.2% | 10.6% | 0.81 | 0.84 | 0.02 | -1.22 |
| I felt lonely | 0.8% | 1.6% | 8.6% | 21.0% | 68.0% | 0.51 | 0.85 | -1.86 | 3.5 |
| I felt confident in myself | 6.2% | 12.0% | 26.2% | 37.0% | 18.6% | 0.85 | 0.84 | -0.53 | -0.38 |
| I did things I found rewarding | 12.0% | 13.6% | 19.4% | 40.2% | 14.8% | 0.86 | 0.84 | -0.54 | -0.73 |
| I avoided things I needed to do | 0.2% | 1.4% | 3.2% | 15.6% | 79.6% | 0.46 | 0.86 | -2.66 | 7.97 |
| I felt irritated | 0.2% | 2.0% | 10.2% | 23.2% | 64.4% | 0.65 | 0.86 | -1.47 | 1.57 |
| I felt like a failure | 0.4% | 0.2% | 1.2% | 6.2% | 92.0% | 0.45 | 0.86 | -5.32 | 35.31 |
| I felt in control of my life | 30.8% | 10.8% | 14.8% | 28.8% | 14.8% | 0.85 | 0.84 | -0.05 | -1.49 |
| I felt terrified | 0.8% | 2.8% | 7.4% | 15.2% | 73.8% | 0.7 | 0.86 | -2.11 | 4.14 |
| I felt anxious | 0.4% | 1.6% | 5.4% | 17.2% | 75.4% | 0.66 | 0.86 | -2.29 | 5.55 |
| I had problems with my sleep | 2.2% | 5.2% | 13.0% | 17.0% | 62.6% | 0.58 | 0.86 | -1.45 | 1.24 |
| I felt calm | 24.8% | 5.0% | 12.6% | 39.6% | 18.0% | 0.56 | 0.86 | -0.5 | -1.21 |
| I found it hard to concentrate | 0.0% | 1.0% | 4.0% | 18.0% | 77.0% | 0.58 | 0.86 | -2.18 | 4.76 |

Table S4 DIF analysis on age and sex

|  |  | ***p*-value of**  **the Chi-square test** | | **McFadden R^2^** | |
| --- | --- | --- | --- | --- | --- |
| item |  | M1 vs. M2 | M2 vs. M3 | pseudo12.  McFadden | pseudo23.  McFadden |
| Negative factor |  |  |  |  |  |
| Sex |  |  |  |  |  |
| 14 | I felt like a failure | **<0.001** | **0.0002** | 0.0633 | 0.0499 |
|  |  |  |  |  |  |
| Age |  |  |  |  |  |
| 12 | I avoided things I needed to do | **0.0002** | 0.4063 | 0.0229 | 0.0011 |
| 17 | I felt anxious | 0.0175 | **<0.001** | 0.008 | 0.0351 |
| 18 | I had problems with my sleep | **<0.001** | 0.9257 | 0.0191 | <0.001 |
|  |  |  |  |  |  |
| Positive factor |  |  |  |  |  |
| Age |  |  |  |  |  |
| 5 | I felt happy | **0.0013** | 0.035 | 0.0068 | 0.0029 |
| 15 | I felt in control of my life | 0.9227 | **0.0004** | <0.001 | 0.0084 |

Note: significant result of M1 vs. M2 indicate uniform DIF; significant result of M2 vs. M3 indicate non-uniform DIF. McFadden’s R2 <0.13 indicates negligible effect size; Bold value indicates significant results


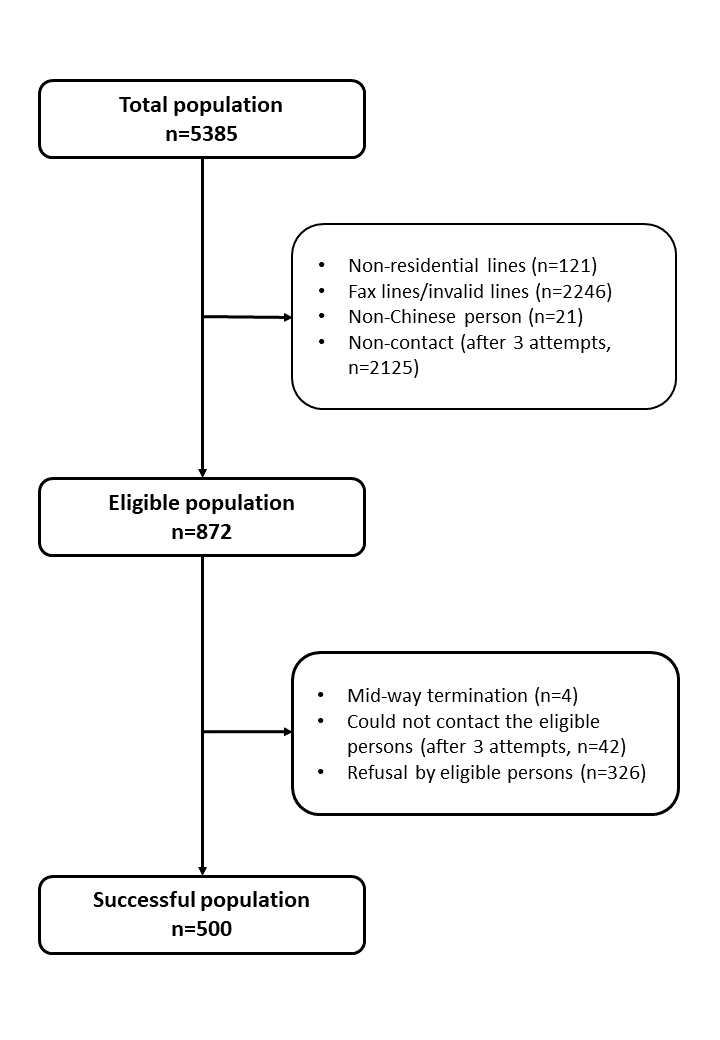


Figure S1 Flowchart of the participant recruitment and selection process


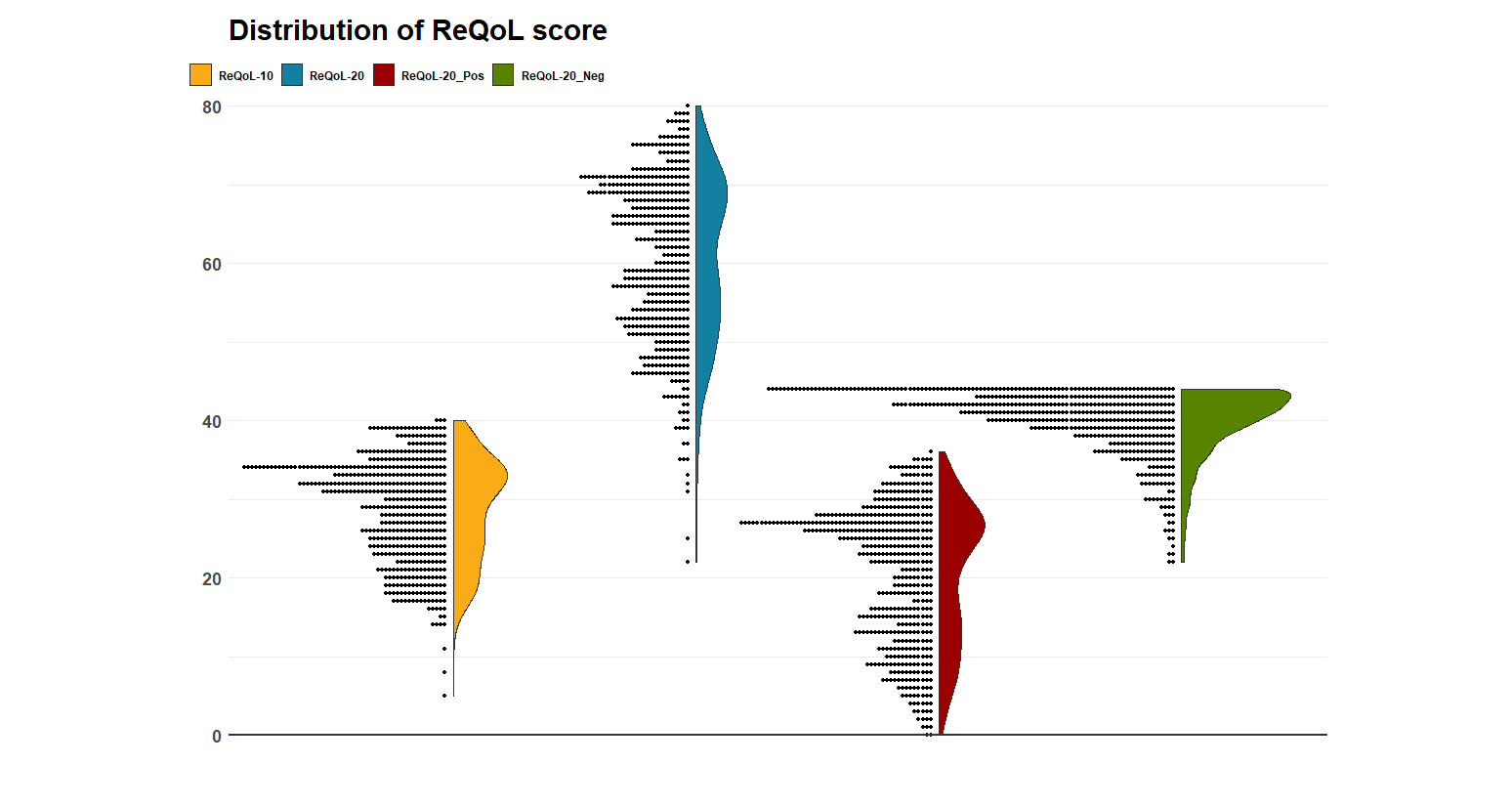


Figure S2 Distribution of sum score of ReQoL measures and stratified according to factor
